# The Calcium Sensor CBL-CIPK Is Involved in Plant's Response to Abiotic Stresses

**DOI:** 10.1155/2015/493191

**Published:** 2015-10-01

**Authors:** S. M. Nuruzzaman Manik, Sujuan Shi, Jingjing Mao, Lianhong Dong, Yulong Su, Qian Wang, Haobao Liu

**Affiliations:** ^1^Key Laboratory of Tobacco Biology and Processing, Tobacco Research Institute of CAAS, Ministry of Agriculture, Qingdao 266101, China; ^2^Chinese Academy of Agricultural Sciences, Beijing 100081, China; ^3^Qingdao Agricultural University, Qingdao 266109, China

## Abstract

Abiotic stress halts the physiological and developmental process of plant. During stress condition, CBL-CIPK complex is identified as a primary element of calcium sensor to perceive environmental signals. Recent studies established that this complex regulates downstream targets like ion channels and transporters in adverse stages conditions. Crosstalks between the CBL-CIPK complex and different abiotic stresses can extend our research area, which can improve and increase the production of genetically modified crops in response to abiotic stresses. How this complex links with environmental signals and creates adjustable circumstances under unfavorable conditions is now one of the burning issues. Diverse studies are already underway to delineate this signalling mechanism underlying different interactions. Therefore, up to date experimental results should be concisely published, thus paving the way for further research. The present review will concisely recapitulate the recent and ongoing research progress of positive ions (Mg^2+^, Na^+^, and K^+^), negative ions (NO_3_^−^, PO_4_^−^), and hormonal signalling, which are evolving from accumulating results of analyses of CBL and CIPK loss- or gain-of-function experiments in different species along with some progress and perspectives of our works. In a word, this review will give one step forward direction for more functional studies in this area.

## 1. Introduction

Unlike animals, plants are not mobile organism and cannot go away from adverse environmental conditions. Owing to these reasons, they create special system to adjust themselves in external stress conditions through instant transmit signals. Due to the temporary fluctuations in cytosolic calcium concentration, plant cells receive the signals from external stimuli, so they can accept the signals using their own machineries and decode the signals to secondary messenger [1–4]. Calcium is broadly well known as a ubiquitous secondary messenger because of its diverse functions in plants. Ca^2+^ is encoded in various stimuli of abiotic and biotic stresses. Abiotic stresses caused by high magnesium, high sodium, low potassium, low phosphorus, ABA, and others affect the rate of germination, photosynthesis, seedling growth, leaf expansion, total biomass accumulation, and overall growth effects of plants [5, 6].

In recent decades, Calcineurin B-like (CBL) protein-CBL-interacting protein kinase (CIPK) complex is widely accepted as Ca^2+^ signalling mechanism, which is involved in response to different external stresses signals [5, 7]. In adverse stresses conditions, plants evolve a stress signal that is specifying Ca^2+^ signature [8–10]. The specific Ca^2+^ signatures are received by closely controlled activities of plasma membrane and other organelles channels and transporters [1, 10–12]. In addition, this signature binds to EF hands domains of the CBL proteins. Consequently, the CBL proteins bind the NAF/FISL domain of C-terminal of the CIPK, thus stimulating the kinase [13]. On the other hand, N-terminal of the CBL protein directs the CBL-CIPK system to an exact cellular target region ensuing in the stimulated CIPK phosphorylating the proper target proteins [11, 14–17].

Bioinformatics and comparative genomic analyses in plants have provided details about the sequence specificity, conservation, function and complexity, and ancestry's history of CBL and CIPK proteins families from lower plants to higher plants. Bioinformatics research reports showed that* Arabidopsis thaliana* has 10 CBLs and 26 CIPKs [13] while in other plants* Populus trichocarpa* has 10 CBLs and 27 CIPKs [18],* Oryza sativa* has 10 CBLs and 31 CIPKs [19],* Zea mays* has 8 CBLs and 43 CIPKs [19],* Vitis vinifera* has 8 CBLs and 21 CIPKs [20],* Sorghum bicolor* has 6 CBLs and 32 CIPKs [20], and* Nicotiana sylvestris* has 12 CBLs and 37 CIPKs (unpublished). Recently some reviewers have focused on functions, structural features, gene expression, and regulation of the CBL-CIPK complex with different pathways [20–24]. Although some reviewers have described the mechanisms, functions, and interaction between the CBL and CIPK, their functional mechanism and regulation with calcium are yet unclear. There is still a huge need to synthesize and understand ongoing findings from current CBL and CIPK studies, so that signalling systems research can be fully harnessed [5, 25–27]. This review will briefly present underlying mechanism of the CBL-CIPK in response to different environmental stresses with emphasis on important pathways. Indeed, it will recap the recent discoveries of these signalling components along with ongoing research progress.

## 2. CBL-CIPK Signalling System Responses to Environmental Stresses

Mutants studies of* Arabidopsis* have demonstrated that the CBL-CIPK complexes are involved in mediating Ca^2+^ signals elicited by different stresses, such as low magnesium, low potassium, high salt, nitrate, low phosphorus, ABA, high pH, cold, and osmotic stress [4, 14, 15, 28–32]. Crosstalk between the CBL-CIPK network and other pathways can limit the distances of improving the tolerant crops in adverse conditions. Different pathways like Mg^2+^, Na^+^, K^+^, NO_3_^−^, PO_4_^−^, and ABA are now burning issues for abiotic stresses. Overexpressing the CBL/CIPK complex in plants might develop their tolerance to concurrently occurring different abiotic stresses and enhance the yield [33]. This complex can posttranslationally phosphorylate its downstream target proteins like transcriptional factors and nutrient pathway to respond to different external environmental stimuli, and thus plant can adapt to unfavorable condition.

To date, research on the CBL-CIPK system has shown that influx/efflux mechanisms of different ions are involved to create an adjustable condition under unfavorable stages in cell. Next session will briefly discuss the mechanism of different pathways.

### 2.1. Magnesium Signalling

Maintaining Ca^2+^/Mg^2+^ homeostasis is not only critical for sufficient supply of mineral nutrients [34] but also important for serpentine-tolerant plants [35].

Recently, a new function has been identified for the CBL-CIPK signalling network in vacuole-mediated detoxification of high external Mg^2+^ [36]. Analysis of double mutant functions of CBL2 and CBL3 (*cbl2-cbl3*) revealed that they are regulating vacuole-mediated Mg^2+^ ion homeostasis in cell [36]. The* cbl2-cbl3* double mutant was hypersensitive to high concentrations of external Mg^2+^ condition, and also ionic profiles analysis showed that a reduced amount of Mg^2+^ accumulation was found in the* cbl2-cbl3* double mutant plants. Tang et al. found that CIPK3/9/23/26 physically interacted with the CBL2/3 on the tonoplast, and the multiple* cipks 3/9/23/26* mutant could fully show hypersensitivity of Mg^2+^, and a similar ionic profile was found as like as the* cbl2-3* mutant [36, 37]. These results strongly suggested that the CIPK3/9/23/26 work together with the CBL2/3 at the tonoplast to alleviate the toxic effects of external high Mg^2+^ concentrations via vacuolar sequestration, but it is not clear which pairs of CBL-CIPK play a vital role in this pathway (Figure 2) [36].

Transporter family AtMHX was the first identified plant Mg^2+^/H^+^ antiporter localized on the tonoplast, which apparently contributes to vacuolar Mg^2+^ uptake [38], and also MGT2 and MGT3 are known as Mg^2+^ transporters localized on the tonoplast [39], but mutant results did not show significant phenotypic changes under high Mg^2+^ conditions [36]. Thus there is further identification of the transporters which are activated under Mg^2+^ toxicity conditions, which are a key step to understand the underlying mechanism of this ion detoxification in plants.

### 2.2. Sodium Signalling

The salt overly sensitive (SOS) pathway is the first identified CBL-CIPK pathway for maintaining ion homeostasis in plant cells [40]. Genetic and biochemical tactics with SOS mutants presented a molecular mechanism in which the CBL-CIPK complex mediates the salt stress-induced Ca^2+^ signal and shows tolerance to salt [41]. Under salt stress situation, this pathway can enhance salt tolerance in plant by multiple ways; for example, it can allow transporter to send back Na^+^ into soil, sequester sodium ion into vacuole, or transport it to the older leaves [24]. The SOS pathway is mainly based on SOS3 (AtCBL4), SOS2 (AtCIPK24), and the plasma membrane Na^+^/K^+^ antiporter; SOS1, a combined component pathway, plays a vital role in effluxing Na^+^ from the cell through SOS1; thus it can enhance the salt tolerance of plants [40]. In salt stress condition, plants can form SOS3-SOS2 complex in their roots and permit the SOS2 to phosphorylate and activate the SOS1 [40]. If plants are unable to activate SOS1 (such as* sos3* mutants), which can store extra Na^+^ through a reduced efflux capacity, thus they inhibit growth under salty conditions [14].

**Figure 1:**
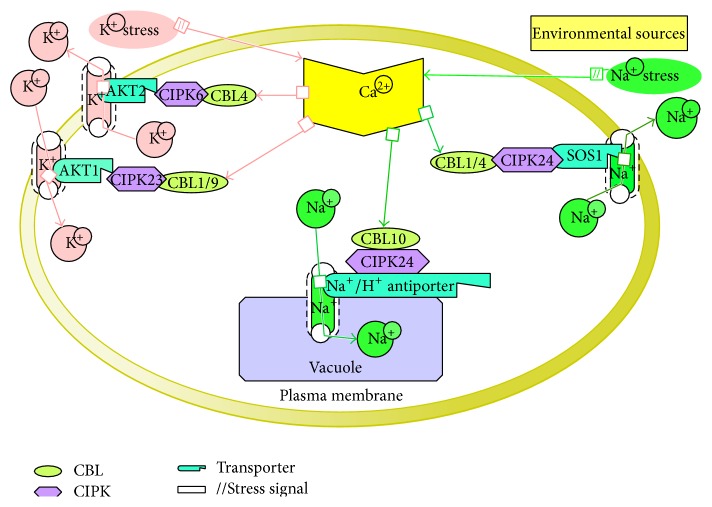
This model represents the identified CBLs-CIPKs interactions process and how they respond to abiotic stresses from environmental sources and maintain homeostasis in cell. All signals are centrally controlled by Ca^2+^. Different colors indicate different pathways. AKT1:* Arabidopsis* K^+^ transporter 1, AKT2:* Arabidopsis* K^+^ transporter 2, and SOS1: salt overly sensitive 1. Mechanism in short: environmental stresses trigger Ca^2+^; Ca^2+^ transmits signal to sensor molecule Calcineurin B-like (CBL) protein-CBL-interacting protein kinase (CIPK) to activate the transporters to create ion homeostasis in cell.

Different CBLs can interact with the CIPK24 and therefore form a complexity system in response to salt stress. External salt stresses trigger the AtCBL4/SOS3-AtCIPK24/SOS2 complex to stimulate Na^+^/H^+^ exchange activity of the SOS1 (Figure 1) [42], which can exclude cell from extra Na^+^ [40].* AtCBL10*, one of the CBL family members, was later included in the salt tolerance pathway. It is thought that tonoplast Na^+^/H^+^ NHX antiporters are activated by the AtCIPK24/SOS2 through a mechanism related to the AtCBL10 to sequester intracellular extra Na^+^ in the vacuole (Figure 1) [43]. Moreover, both CBL4/SOS3 and CBL10 are involved in mediating salt tolerance, but they perform their functions in different ways because of their distinct subcellular localizations and expression pattern.

**Figure 2:**
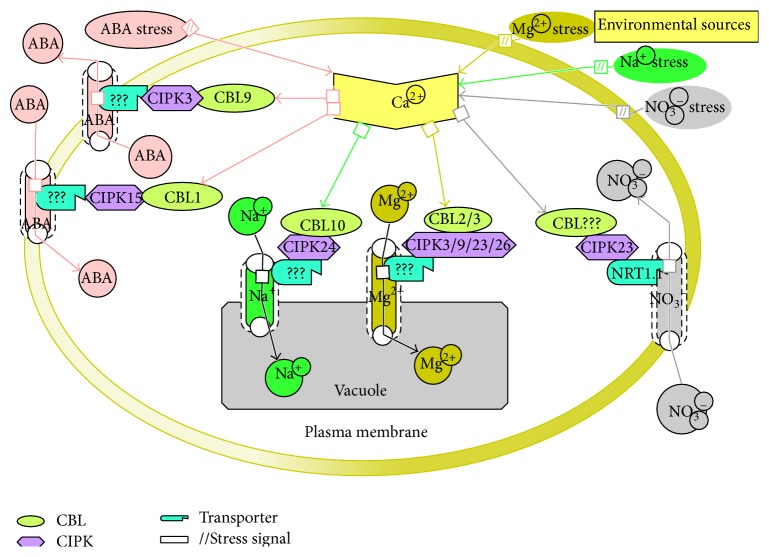
This model represents partially identified CBLs-CIPKs signalling system. Here, question marks (???) indicate that components have not yet been identified. Different colors indicate different pathways. NRT1.1: nitrate transporter 1.1.

Tissue specific and subcellular localization experiments showed that the CBL4/SOS3 works primarily in the roots and is localized at the plasma membrane, respectively [40]. Thus the CIPK24/SOS2 functions at the same place where it phosphorylates Na^+^/H^+^ antiporter SOS1, thereby enhancing Na^+^ efflux rate [40]. Compared with the CBL10, it is expressed predominantly in the shoots and leaves and localized at the vacuolar membrane (tonoplast) [44]. It is postulated that the CIPK24/SOS2 employed by the CBL10 on the tonoplast may phosphorylate and activate as a yet unknown Na^+^ channel or transporter, which is the tonoplast bound and performs a role in transporting cytoplasmic Na^+^ into the vacuolar space (Figure 2). That assumption is supported by knockout* Arabidopsis* mutant* cbl10*, which showed the salt-sensitive phenotype specifically in the leaves or shoots and accumulated less Na^+^ than the wild type under high salt conditions [44].

Additionally, other studies have shown that a calcium sensor, CBL1, can also interact with the CIPK24 to mediate the regulation of Na^+^ in the plant cell (Figure 1) [13]. Thus* cbl1* mutant plants showed less tolerance to salt stress [45]. Subcellular localization assay demonstrated that the CBL1 is localized in the plasma membrane and interacted with the CIPK24/SOS2 as the CBL4/SOS3, and expression pattern analysis showed that it is expressed in the shoots and roots [45]. So, it can be said that Na^+^ extrusion mediated by the CIPK24/SOS2-SOS1 system may also occur in the shoots.

Not only do CBLs show the salt sensitivity but also CIPKs are sensitive to salinity conditions.* Arabidopsis cipk6* was described to be more sensitive to salt stress compared to the wild type and it is thought that CIPK6 might be involved in salt tolerance [46]. Interaction between the CIPK6 and the CBL4/SOS3 was proved by yeast two-hybrid system, which indicated the participation of the CIPK6 in this pathway [16]. Possibly, the CBL4/SOS3 also targets the CIPK6 in vivo as well as the CIPK24/SOS2. Further research can shade more light on this complex mechanism involved in response to salt stress.

Apart from the experiments on* Arabidopsis*, recently, researchers have done some experiments on other species and tried to understand the salt pathway clearer. For instance, apple MdCIPK6L-OE conferred tolerance to salt [47] and its ectopic expression could functionally complement* Arabidopsis sos2* mutant, even though it was not homologous to the* Arabidopsis* CIPK24/SOS2 [47]. Besides MdCIPK6L-OE,* MdSOS2* was cloned from apple, which showed the highest similarity to the AtCIPK24/SOS2, and also it positively responds to salt stress and functionally complements the* Arabidopsis sos2* mutant [48]. The structural and functional analysis of BjSOS3 was established in the SOS pathway in* Brassica juncea* [49]. In rice OsCBL4 was the most homologous to the AtCBL4/SOS3 and it was able to functionally complement* sos3-1* mutant in* Arabidopsis*, indicating that it has the same function as the AtCBL4/SOS3 [50]. ZmCBL4 is the most similar to the OsCBL4 and it can also complement the* sos3-1* mutant in* Arabidopsis* [51]. In* Nicotiana sylvestris* CBL10 also showed salt sensitivity in* Arabidopsis*, which demonstrated more tolerance phenotype than wild type Colombia plants under salt stress condition (unpublished). Among the identified CBLs and CIPKs in response to salt stress, only a few have been implicated as negative regulators of salt pathway. For example, AtCBL1 and poplar (*Populus euphratica*) PeCBL1 were found to negatively influence Na^+^ efflux from the cell under saline conditions while the mechanisms behind this are still unclear [52].

### 2.3. Potassium Signalling

Potassium (K^+^) is one of the most important mineral nutrients, which participates in various plant physiological processes and governs yield of crop production. Plants recognize external K^+^ fluctuations and create preliminary K^+^ signal in root cells [53]. Root cell then transfers signals into cytoplasm, which signals are sensed by calcium sensors [53]. Since 1992, AKT1 is called a low affinity inwardly rectifying K^+^ channel, which is involved in the cellular uptake of K^+^ signal via calcium sensors [15, 31, 32, 55]. The calcium sensor CBL-CIPK acts as a regulator of the AKT1 to maintain the homeostasis of potassium in cell [31, 56].

If the amount of external K^+^ became low, one of the CIPKs, CIPK23, is targeted to the plasma membrane, which is concurrently stimulated by CBL1 and CBL9 to phosphorylate the AKT1; thus movement of K^+^ will be inwardly into the cells (Figure 1) [15, 31, 32, 56]. Experiments on mutants* cipk23*,* cbl1/cbl9*, and* akt1* showed similar reduced growth and chlorotic leaves under low K^+^ conditions [15, 32, 36, 57]. It is hypothesized that the* cbl1/9* are functionally overlapped, because they individually did not show any significant differences. But their tissue specific localization assay demonstrated that they are expressed in root cells and aerial tissues, such as guard cells and vascular cells as like as localization of the AKT1 [15, 32]. Although the AKT1 expressed low level in hydathodes and stomatal guard cells, the AtCIPK23 may be regulated by the AtCBL1 or AtCBL9 in aerial tissue to redistribution of K^+^, turgidity of guard cell, and repolarization of cell membrane [55, 58–60]. Instead of mutant experiments, AKT1 overexpressed (OE)* Arabidopsis* plants did not show any significant performance in growth when they were grown in low K^+^ conditions, while At/PeCBL1, AtCBL9, and AtCIPK23 OE* Arabidopsis* plants gave comparative tolerance compared to control plants under the same condition [61, 62]. Recently overexpressed AtCIPK23 in potato [63], coexpression of AtCBL9-AtCIPK23-AKT1 in sugarcane [63, 64], OsCBL1-OsCIPK23-OsAKT1 in rice [64], VvCBL1-CIPK4-VvKT1.1 and VvCBL2-CIPK3-VvKT1.2 in grapevine (*Vitis vinifera*) [65] showed improved tolerance under the low potassium conditions. Moreover, the activity of AKT1 can be negatively regulated by a PP2C-type phosphatase AKT1-interacting PP2C1 (AIP1) [56]. Therefore, the CBL1/CBL9-CIPK23 complex can phosphorylate and activate the AKT1, but dephosphorylation by the AIP1 may regulate the deactivation of the AKT1 [56].

Another study showed that CBL4 interacts with CIPK6, so CBL4-CIPK6 complex is controlling the plasma membrane targeting of the* Arabidopsis* K^+^ channel AKT2 by facilitating translocation to the plasma membrane (Figure 1) [16]. In addition, alone the regulatory C-terminal domain of CIPK is sufficient to mediate the CBL4- and Ca^2+^-dependent channel translocations from the ER membrane to the plasma membrane [66]. This interaction system of the CBL4 is accomplished through a unique targeting pathway that is dependent on the dual site (myristoylation and palmitoylation) [16]. Thus this is a unique system designated as a critical mechanism of ion-channel regulation, in which a calcium sensor controls K^+^ channel activity by promoting the translocation of the channel to the plasma membrane [66] that is together in kinase interaction-dependent and phosphorylation-independent manner [16]. These studies suggest that the* Arabidopsis* K^+^ channel AKT2 proficiently translocates to the plasma membrane through the CBL4- and Ca^2+^-dependent targeting pathway that entails the scaffolding task and the kinase activity of the CIPK6. This is consistent with the hypothesis that there are multiple pathways for K^+^ channel operating. Besides, CIPK9 responds to various abiotic stresses, such as salinity, osmotic stress, chilling, and cellular injury, and also it plays a critical role in plant tolerance to low K^+^ [67]. The knockout T-DNA mutant lines of* cipk9* displayed a hypersensitive response to low K^+^ conditions. However, further analysis specified that K^+^ uptake and content were not affected in the mutant plants [67]. It has been inferred that the* Arabidopsis* CIPK9 might have a different mode of action than the CIPK23 and CIPK6. It is possible that unknown CBLs interact with the CIPK9 to regulate K^+^ homeostasis by activating a vacuolar potassium channel [68]. It can also be hypothesized that the unknown CBLs may interact with different CIPKs to sense Ca^2+^ signals in low K^+^ stress conditions [68]. Indeed, there is still needed further research to qualify this assumption.

### 2.4. Nitrate Signalling

Nitrogen is a key limiting element for crop production and overall plant growth. NO_3_^−^ form of nitrogen, which is the principal nitrogen source of plants [69], research on NO_3_^−^ uptake system, provides a test case to define the nutrient transport system to unravel plant nutrient acquisition signalling pathways. However, the molecular mechanisms of NO_3_^−^ sensing and signalling have just started to be unraveled in* Arabidopsis thaliana*. The members of three nitrate transporter families, such as 53 of AtNRT1, 7 of AtNRT2, and 7 of AtCLC, have been identified in this plant [70–72]. Among the three families, four plasma membrane transporters members of AtNRT1 and AtNRT2 families are occupied in uptake of NO_3_^−^ by root cells [72, 73]. Members of AtNRT2.1 and AtNRT2.2 are engaged in high-affinity uptake that drive either a high affinity (nitrate concentration < 1 mM) or a low affinity (nitrate concentration > 1 mM) [74, 75], and AtNRT1.2 is worked in low-affinity uptake whereas AtNRT1.1 (CHL1) is performed as a dual-affinity transporter involved in both high- and low-affinity uptake of NO_3_^−^ [76, 77].

The CHL1 functions as a high-affinity nitrate transporter when threonine residue 101 (T101) is phosphorylated and as a low-affinity nitrate transporter when this residue is dephosphorylated [78, 79]. The first report of a potential role for the CHL1 in nitrate signalling originated from the studies of loss-of-function mutant (*chl1*) in* Arabidopsis*, which demonstrated that the CHL1 regulates the expression of AtNRT2.1 in response to nitrate stress [77]. In microarray system, it showed that AtCIPK23 was downregulated in the* chl1* mutant (Figure 2). However, the AtCIPK23 is not only the target of the AtNRT1.1-dependent signalling but also a regulator of the AtNRT1.1, which is responsible for its phosphorylation at the T101 residue [78]. The AtCIPK23 therefore governs both transport and signalling activities of AtNRT1.1, which infers that the incidence of retrocontrol loop for the AtNRT1.1-dependent gene acts in response to NO_3_^−^. Remarkably, the mechanisms leading to the AtCIPK23-mediated phosphorylation of the CHL1 are required to fully understand the possible role of the CHL1 in direct sensing of external nitrate.

In addition,* Arabidopsis* CBL9 is required to activate the AtCIPK23 to mediate the phosphorylation of CHL1 for high-affinity nitrate transportation but the activity of this signalling system remains obscure [80]. Transcriptomic study presented that* Arabidopsis* CIPK8 is involved as a low-affinity nitrate response under stress conditions [81]. Results of continuous experiments on* cipk8* mutant lines showed that the AtCIPK8 is involved in long-term nitrate-regulated root growth and it positively sets the primary nitrate response. In short, the* Arabidopsis* CIPK8 precise regulation of AtNRT1.1 is still unclear and needs further analysis [81].

### 2.5. Phosphorus Signalling

Phosphorus is known as a secondary macronutrient in plant [82]. Pi (inorganic phosphorus) form of phosphorus is readily absorbed by plants in phosphorous deficient condition [83]. Pi is involved in controlling major enzymatic reactions and switching the metabolic pathways [84]. A report by Chen et al. has published that the CBL-CIPK system is involved during the response to low Pi in* Brassica napus*. Under Pi deficient conditions, BnCBL1 and BnCIPK6 were upregulated and both proteins can interact with each other in yeast two-hybrid screens and split-YFP system [85]. Under low Pi treatment, overexpression of either BnCBL1 or BnCIPK6 showed better plant growth and accumulated more biomass in* Arabidopsis*, mostly found in the lateral roots development [85]. So, the BnCBL1 and BnCIPK6 might control the processes involved in the plant's response to Pi deficiencies, even though the mechanism and pathways are still unknown. It is not clear whether AtCIPK6 is involved in low Pi pathway, though the complementary experiment of the BnCIPK6 with* cipk6* mutant showed that it also responded to low Pi treatment. There is still need for further research in this area [57, 85].

## 3. Hormonal Signalling

Abscisic acid (ABA) is one of the most essential phytohormones in plants. It performs different roles in plants ranging from seed germination to growth and development as well as responses to abiotic stresses [86]. A specific Ca^2+^ signature responder is found in an early step of the ABA signalling pathways system [87–89], which implies that Ca^2+^ sensors are involved in this signalling pathway. Moreover, studies on several overexpressed/mutant lines of CBL/CIPK inferred that the CBL-CIPK system is involved in the ABA signalling pathway (Figure 2).

Although the ABA signalling pathways are mainly regulated by two ways, such as ABA-dependent and ABA-independent ways, which are simultaneously controlled stress-responsive genes, ABA-dependent pathway shows a vital role in regulating osmotic stress-responsive genes [90]. The* Arabidopsis* mutant plants lacking CBL9 (*cbl9*) displayed hypersensitivity to ABA in the early developmental stages, such as seed germination and postgermination seedling growth [4]. Experimental results also showed that the* cbl9* accumulated much higher levels of ABA than the control plants under stress conditions [4]. Therefore, the AtCBL9 performs as a negative regulator in abscisic acid signalling [4]. Besides, the expression of AtCIPK3 is induced by cold, high salt, wounding, drought, and ABA. Seed germination analyses of* cipk3* mutants indicated that these lines were more inhibited by the ABA than wild type plants, and results indicated that the AtCIPK3 functions as a negative regulator in ABA signalling during seed germination [91]. It was also demonstrated that the AtCBL9 can form a specific complex with the AtCIPK3 to act together in regulating the ABA responses [92] and suggesting that the AtCBL9-CIPK3 complex negatively regulates the ABA signalling during seed germination (Figure 2) [92].

Furthermore, CBL1 is the most similar isoform of the CBL9 in* Arabidopsis*. Evaluation of the CBL1 function based on loss-of-function mutant showed that (*cbl1*) lines are hypersensitive to abiotic stresses [28, 45]. The* cbl1* did not show significant changes in response to the ABA, but CBL1 and CBL9 mutant lines both displayed less tolerance to drought and salt stress [28, 45]. These results indicated that the CBL1 is not involved in the ABA signalling system dissimilar to the CBL9. Meanwhile, it is remarkable to note that CIPK1 can interact with the CBL1 and CBL9, which mediates ABA responses as well as osmotic stress, drought, and salt responses. Above those factors infer that CBL1-CIPK1 complex is involved in the ABA-dependent way; however CBL9-CIPK1 complex is occupied in the ABA-independent way in* Arabidopsis* [29]. One more research informed that knockdown* cbl1* and* cipk15* generated an ABA-hypersensitive phenotype [93]. Thus CBL1-CIPK15 complex works as a negative regulator in the ABA signalling pathway (Figure 2) [93]. A recent study found that CIPK6 loss-of-function lines (*cipk6*) accumulated high level of ABA in seedlings after treatment, compared to the primary level of expression. This finding implies that the CIPK6 is also involved in responses to ABA [46].

Very few reports have been published of interaction between GA and CBL-CIPK. Research showed that rice CBL gene OsCBL2 was upregulated by gibberellin acid in the aleurone layer in rice [94]. It also showed that this CBL is positively regulating the GA pathway. Using microarray analyses and RNA blots, they have found that the upregulation of the OsCBL2 expression occurs within specific time period after GA treatment [94]. Taken together, these data indicate that CBL-CIPK system plays an important role in the hormonal signalling pathway.

## 4. Conclusions and Perspectives

Studies on CBLs and CIPKs over the past few years have greatly advanced our knowledge of the function of single proteins in distinct physiological processes. Major advances in our understanding of this signalling system have been made possible by the identification of an increasing number of targets regulated by the CBL-CIPK complexes.

The unraveling of the crosstalk among different pathways will provide more information about the physiological responses of plants, including transpiration, germination of seeds, seedlings growth, and uptake of mineral nutrient under different stress conditions. The progress of the research on the CBL and CIPK families in different plant species other than* Arabidopsis thaliana* is still at an infant stage; in most cases it is limited to interaction studies and expression analyses of these families. Recently, some experiments have been done on the CBL-CIPK complex on poplar, rice, pea, and maize [27]; which experiments indicate an overall participation studies of the research on CBL-CIPK in responses to different abiotic stresses. A few members of the CBLs and CIPKs from above species have been functionally identified, and expression profile has been done in response to stresses, such as salt, drought, cold, and plant hormones [50, 51, 95–98].

Future research should put emphasis on identifying further signalling components over a period generation of mutants by gene knockout approaches and subsequent dissecting of gene functions. Fascinating new insights and prospects are emerging as a result of the increasing number of available genome sequences, which will assist the investigation of the ancestries and functional diversification of these calcium sensors and their interacting protein kinases into the extant complex interaction network. The mechanisms conferring this complex interaction specify the regulatory capabilities to rely on the intermolecular interactions between CBLs and CIPKs [99]. The CBL-CIPK signalling model emphasizes the importance of future research that focuses on the molecular mechanisms underlying the regulation of transporters that allow us to better understand plant's response to abiotic stress and also establish a proficient method of identifying molecular targets for genetically engineered resistant crops with enhanced tolerance to various environmental stresses. Therefore, the most important challenge for future research is not only functional thesis but also the elucidating of the details of synergistic functions in this interaction network and revealing of the molecular mechanisms of the complexes regulating target proteins.
